# Nurses’ stressful experiences in providing palliative and end-of-life care for children: A meta-synthesis

**DOI:** 10.1017/S1478951526102880

**Published:** 2026-07-20

**Authors:** Ratna Puji Priyanti, Asri, Yu-Wen Chen, Shih-Chun Lin

**Affiliations:** 1 School of Nursing, College of Nursing, Kaohsiung Medical University, Kaohsiung, Taiwan; 2 School of Nursing, Stikes Pemkab Jombang, Jombang, Indonesia; 3 Faculty of Health Science, Muhammadiyah Surabaya University, Surabaya, Indonesia; 4 Center for Long-Term Care Research, Kaohsiung Medical University, Kaohsiung, Taiwan; 5 Department of Medical Research, Kaohsiung Medical University Hospital, Kaohsiung, Taiwan

**Keywords:** Children, nurse, palliative care, death, stress

## Abstract

**Objectives:**

To explore the stress experienced by nurses while addressing the needs of families during palliative and end-of-life care for children and young adults.

**Methods:**

A meta-synthesis was conducted. Six online databases (PubMed, Medline, EMBASE, Cochrane Library, CINAHL, and Airiti) from 2014 to September 15, 2025 were searched.

**Results:**

Of the 1,841 citations identified, 34 qualitative studies and 12 case reports were included. All included studies had adequate to strong methodological quality. Three themes emerged from the qualitative findings: (1) never seem to feel fully prepared; (2) sense of unfairness in conflicts of values; and (3) sheathing against traumatic bond attachment.

**Significance of results:**

Nurses experience various stresses throughout the nursing process, which evoke avoidance responses. Supporting nurses is as crucial as providing family-centered palliative care to ensure a sustainable healthcare workforce. These findings demonstrate the importance of understanding how nurses perceive their stress in relation to provision of pediatric palliative care to help develop personal and team resilience.

## Introduction

Providing palliative care for children required an emotionally demanding experience for both family members and nurses, often resulting in significant emotional strain. These conditions, characterized by the anticipation that a child will not reach adulthood, demand continuous, complex, and often intensive care (Dryden-Palmer et al. 2018). Nurses are responsible for conducting a comprehensive assessment of the family members to understand their daily functioning and caring needs for their child. However, this process also involves a burden of shared responsibility of providing care, support for the family with a life-limited child, and a commitment to ensuring accessibility for the family on a continuous basis.

Families face numerous challenges when caring for their critically ill children, including providing support and nurturing (Dryden-Palmer et al. 2018). The loss of a child can lead to long-term grief and significantly impact family members’ lives (Lin and Huang 2020). Nurses play a crucial role in addressing the grief of these families by offering palliative care (Lin and Huang 2020). Nurses are expected to facilitate child-centered care through conversations about what matters most to the patient, considering each child’s unique losses and experiences. This approach allows for a more comprehensive understanding of patients’ preferences and priorities (Coombes et al. 2023; Bristowe et al. 2024). For instance, some families are concerned that palliative care or death issues may bring despair and threaten their child’s and the family’s hope for the child’s recovery (Lin and Huang 2020). Uncertainty in terms of the prognosis and the benefits of treatment, with the goals of care swinging between extending life and alleviating suffering, have also been reported as reasons for nurses to delay raising the palliative care issue (Kaasa et al. 2018; Lin et al., 2022). Many cultural minorities seek for more optimistic and hopeful information from spiritual sources when facing end-of-life decision-making (Lin and Huang 2020), which may concern nurses attempting to initiate palliative care (Lin et al. 2022). Therefore, their needs span a wide spectrum, from emotional support and clear communication from healthcare providers to practical assistance and respite care (Gill et al. 2021). Addressing these needs is vital for maintaining family stability and ensuring the child’s holistic well-being.

Nurses at the forefront of delivering care often experience significant stress. This stress stems from the emotional burden of providing palliative and end-of-life care (Gill et al. 2021), the physical demands of the job, and the high-stakes decision-making required in managing complex cases. Despite these challenges, nurses demonstrated their remarkable resilience and ability to provide quality care. Nurses deliver compassionate and skilled medical attention while simultaneously managing their emotional well-being. However, chronic stress among nurses can lead to burnout, compassion fatigue, and decreased job satisfaction, which can significantly impact patient care quality (Xie et al. 2021).

Previous research has shed light on the barriers for families and professionals to referral to palliative and end-of-life care for children (Lin et al. 2022). However, there is a pressing need for a deeper understanding of how familial needs intersect with the stress experienced by nurses providing direct care. Nurses’ stress in providing pediatric palliative care is a significant concern as they stand a role to promote patients’ and families’ quality of life and reducing health-related suffering, especially at the end of life. Yet, the intention to meet families’ needs, providing pediatric palliative and end-of-life care context, has an effect on nurses’ stress that is not clearly established. The method pediatric nurses provide professional care for families of children with palliative care needs, especially in the absence of specialist palliative care involvement or when nurses themselves are grappling with moral distress, remains a grey area. This interplay between parental demands and nurses’ emotional burden has profound implications for the quality of care delivered and the overall well-being of all parties involved, making this research critically important to our audience.

In this study, we use a critical realist paradigm to analyze the influence of the intention or pursuit of meeting families’ needs on nurses’ stress. Specifically, we draw from the theory of families’ needs to identify the expectations and preferences of family members for the care of children with palliative care needs, to understand nurses’ efforts or ideal circumstances for meeting these needs, and further to draw from a meta-synthesis to identify related nurses’ stress. By developing a deep understanding of this intricate dynamic, the research seeks to inform strategies that improve support systems and resources available to both families and nurses, providing much-needed relief and support in their challenging journey. This study aims to explore the stress that nurses experience while meeting the needs of families during palliative and end-of-life care for children and young adults. To achieve this, a systematic review and meta-synthesis will be conducted, following the guidelines set out by the Preferred Reporting Items for Systematic Reviews and Meta-Analyses (Page et al. 2021).

## Methods

### Design

A meta-synthesis was conducted using a critical realism approach, and Thomas and Harden’s (2008) thematic analysis framework guided the analysis.

### Inclusion and exclusion criteria

The review included English or Mandarin-language qualitative studies of any design, mixed-method studies, where qualitative data can be exclusively extracted, or case reports of nursing experiences. Exclusion criteria were studies focusing on patients outside the 0–25 age group; combining care for patients outside the specified age group and could not be separately analyzed; non-palliative care or end-of-life care; and not nursing perspectives. EndNote Version 20 was used for organizing and removing duplicated search results.

### Search strategy

Data collection for meta-synthesis involved searching online databases, including MEDLINE, EMBASE, PubMed, Cochrane Library, CINAHL, and Airiti (a Chinese database) from January 1, 2014 to September 15, 2025. The search terms used included the recognized Medical Subject Headings and Embase subject headings (Emtree) with various combinations of the terms nurse, children, palliative care, end-of-life, and stress. The following data from the included studies were extracted by the second author and checked by the first author: primary author, year of publication, the country where the study was conducted; only sections on nurses’ views were included.

### Quality assessment

Two researchers (Y.-W.C. and S.-C.L.) independently assessed the study quality using the Joanna Briggs Institute checklist (Lockwood et al. 2020). The scoring method (2 for yes, 1 for unclear, and 0 for not applicable) was adapted from Lin et al. (2021). Quality is interpreted based on the total score: >80% is strong, >70% is good, >50% is adequate, and ≤50% is limited (Lin et al. 2021).

### Data analysis

A thematic synthesis technique (Thomas and Harden 2008) was used to explore the stress experienced by nurses in caring for families’ needs. Our approach is informed by critical realism (Groff 2010; Maxwell 2012), which is characterized by its explanatory goals and reliance on theoretical support (Clark 2008; Groff 2010). Three research team members engaged in the synthesizing process and resolved any disagreement through discussion. The data were analyzed using NVivo 14 software.

### Rigor and reflexivity

The first author (S.-C.L.), who has a background as a pediatric nurse, collaborated with a parent expert who had lost 2 children to life-limiting conditions to ensure a comprehensive interpretation of the findings. Thick descriptions of themes and quotes were provided to increase the transferability of the findings, which allows implementation in other fields, such as patients with chronic conditions, professional-family relationships, and moral distress issues. An audit trail was provided by presenting the research design and process for confirmability (Lincoln and Guba 1985).

## Results

### Characteristics of the included studies

**Figure 1.**
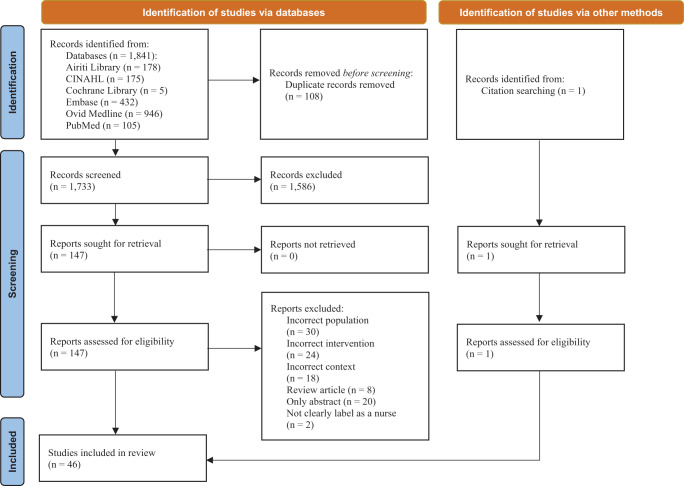
PRISMA 2020 flow diagram. Figure 1 long description.

The initial search yielded 1,841 studies. After the exclusion of 108 duplicates, 1,733 titles and abstracts were screened, and 1,586 were excluded due to irrelevance. Then, 147 articles were further assessed for eligibility, and 46 studies were selected and included in the final synthesis. Out of 46 articles, 34 reported a qualitative research method, and 12 were case reports of nursing experiences (Fig. 1).

All included studies had adequate to strong methodological quality, with scores ranging from 60 to 100%. Table 1 summarizes the study characteristics and quality of the included studies.

**Table 1.** The study characteristics and quality of the included studies Table 1 long description.

First author, year, country	Aim	Study design	Setting	Data analysis	Number of nurse participants studied sample (n)^a^	Quality appraisal
Al Mutair 2019, Saudi Arabia	To explore the perceived impact and influence of aspects of cultural diversity on how NICU/PICU nurses care for Muslim families before and after the death of infants/children.	Qualitative descriptive-exploratory design	NICU and PICU of a large private hospital	Thematic analysis	13 (NICU nurse *n* = 6; PICU nurse *n* = 7)	16/20 (80%), good
Almeida et al. 2016, Brazil	To understand the experiences of nurses when caring for dying newborns and their families in the NICU; and redeem their perceptions about acting before the death and grieving process.	Qualitative descriptive-exploratory design	NICU of a private hospital	Categorical, Collective Subject Discourse method, grounded in the theory of Social Representation	9	17/20 (85%), strong
Atout 2019, Jordan	To explore the experience of disclosing critical information in the care of children with palliative care needs, from the perspective of physicians, nurses, and mothers in Jordan.	Qualitative collective approach	Medical floor/surgical floor/PICU	Within-case and cross-case analysis	21 (head nurse *n* = 1; charge nurse *n* = 2; registered nurse *n* = 17; practical nurse *n* = 1)	17/20 (85%), strong
Avery 2019, Canada	To explore the experiences of HCPs involved in introducing and providing palliative care to adolescents and young adults with advanced cancer and their families to understand the unique challenges healthcare providers experience.	Qualitative descriptive design	2 tertiary hospitals	Thematic and constant comparative analysis	4 (nurse practitioners)	15/20 (75%), good
Banazadeh et al. 2021, Iran	To explore healthcare professionals-related factors affecting parents’ participation in decision-making for neonates with life-threatening conditions.	Qualitative using grounded theory method	NICU of teaching hospitals	Constant comparative analysis	4	16/20 (80%), good
Bassola 2023, Italy	To understand the experience of nurses as caring for infants and children with life-limiting and life-threatening conditions.	Qualitative phenomenological descriptive approach	Pediatric and neonatal wards in a general hospital	Colaizzi method	27 (general nurse *n* = 16; pediatric nurse *n* = 11)	15/20 (75%), good
Bian et al. 2023, China	To explore pediatric nurses’ challenges and effective coping strategies in caring for dying children.	Qualitative descriptive design	Pediatric/pediatric emergency/neonatology departments	Content analysis	10	18/20 (90%), strong
Burgers 2022, Netherlands	To understand the challenges in daily clinical practice experienced by professionals from different disciplines who provide palliative as well as general care to adolescents and young adults with an uncertain and/or poor cancer prognosis.	Qualitative descriptive design	12 hospitals	Reflexive thematic analysis	10 (clinical nurse specialist)	17/20 (85%), strong
Chen 2015, Taiwan	To describe the accumulated nursing experiences from providing palliative care to a premature infant with severe persistent pulmonary hypertension.	Case study	NICU of a medical center	Narrative analysis	1	15/16 (94%), strong
Chen 2019, Taiwan	To explore the difficulties in end-of-life care for cancer children.	Case study	Pediatric ward of a medical center	Inductive, content analysis	6	18/20 (90%), strong
Chen 2021, Taiwan	The author accompanies the family facing the disease progression and the terminal stage, and fulfilling hospice care experience through the coordination with hospice shared-care model.	Case study	Pediatric ward of a medical center	Narrative analysis	1	16/16 (100%), strong
Chew et al. 2021, Singapore	To explore the experiences, challenges, and coping strategies of new nurses dealing with pediatric death in a clinical setting.	Qualitative descriptive design	PICU/high dependency ward/oncology ward	Thematic analysis	12 (PICU nurse *n* = 7; high dependency nurse *n* = 2; oncology nurse *n* = 3)	16/20 (80%), good
Chiu 2018, Taiwan	The paper describes the nursing experience of caring for a neonate with Patau syndrome.	Case study	NICU of a teaching hospital	Narrative analysis	1	14/16 (88%), strong
Coats 2018, USA	To describe nurses’ perceptions of the benefits and challenges of providing family-centered care in PICU.	Qualitative descriptive design	PICU/cardiac ICU/NICU in an urban children’s hospital	Content analysis	10 (PICU nurse *n* = 4; cardiac ICU nurse *n* = 3; NICU nurse *n* = 3)	14/20 (70%), adequate
Constantinou et al. 2024, UK	To qualitatively explore professionals’ lived experiences of providing care to children with life-limiting conditions and their families.	Descriptive phenomenological study	Services in and around Bedfordshire, Hertfordshire and Milton Keynes, UK	Thematic analysis	5 (inpatient unit nurse *n* = 1; children’s community nurse *n* = 1; senior children’s nurse *n* = 1; children’s nurse *n* = 1; clinical nurse specialist *n* = 1)	20/20 (100%), strong
de Medeiros 2022, Brazil	To identify the nursing staff’s perception of their relationship with families of newborns and children who are in the process of death and dying.	Descriptive-exploratory design	NICU and Pediatrics sectors of a maternity hospital	Content analysis within the framework of Interpersonal Relations theory	17 (nursing technicians *n* = 10; nurse *n* = 7)	17/20 (85%), strong
Deschenes et al. 2024, Canada	To explore what pediatric critical care nurses identify as a needed intervention to minimize moral distress.	Descriptive-exploratory design	2 PICUs at a tertiary care hospital	Categorical strategy	10	16/20 (80%), good
Devitt and Hara 2021, Ireland	To explore the perceptions of nurses within the acute healthcare setting who care for children with life-limiting conditions.	Qualitative study	Pediatric wards	Thematic analysis	14	13/20 (65%), adequate
Donohue 2018, USA	To assess healthcare professionals’ experiences of burnout and perceptions of family burnout in caring for children with chronic critical illness.	Qualitative study	Neonatal or pediatric ICUs, other inpatient sites, rehabilitation facilities, outpatient pediatric practices, and home	Content analysis	15 (nurse and nurse practitioner)	15/20 (75%), good
Dos Santos et al. 2023, Brazil	Explore ethical and moral conflicts arising in the field of pediatric oncology from the perspective of nursing professionals.	Descriptive-exploratory design	Inpatient and ICUs of a pediatric cancer hospital	Secondary, thematic analysis	10 (inpatient unit nurse *n* = 3; ICU nurse *n* = 3; inpatient unit nursing technicians *n* = 2; ICU nursing technicians *n* = 2)	15/20 (75%), good
Duarte 2021, Brazil	To analyze the experiences that generate pleasure and suffering in the work of nurses.	Descriptive-exploratory design	Pediatric oncology inpatient unit	Thematic content analysis	8	20/20 (100%), strong
Fernández-Alcántara 2020, Spain	To identify and examine the subjective experiences and practices of experienced professionals attending to perinatal loss in the hospital context.	Exploratory descriptive design	3 public hospitals	Thematic analysis	8 (nurse *n* = 4; nurse assistant *n* = 4)	17/20 (85%), strong
Geoghegan 2016, UK	To gain a deeper understanding of the impact on staff of caring for children who have a prolonged stay on the PICU.	Qualitative study	3 ICUs and adjoining wards in a tertiary pediatric healthcare setting	Framework approach	7	12/20 (60%), adequate
Han et al. 2023, South Korea	To describe nurses’ experiences of moral distress when they provide end-of-life care to infants and their families.	Descriptive design	NICUs in 2 tertiary hospitals	Secondary content analysis based on Corley’s theory of moral distress	20	18/20 (90%), strong
Hsia 2020, Taiwan	To describe the palliative care experience of a brain-dead infant and his parents.	Case study	PICU of a medical center	Narrative analysis	1	12/16 (75%), good
Hsu and Liu 2014, Taiwan	To describe the nursing care experience concerning an infant with head injury and his mother faced with her dying baby.	Case study	NICU of a teaching hospital	Narrative analysis	1	13/16 (81%), strong
Huang 2022, Taiwan	To describe the nursing experience of a neonate with congenital pulmonary airway malformation and pulmonary dysplasia who received extracorporeal membrane oxygenation.	Case study	NICU of a medical center	Narrative analysis	1	13/16 (81%), strong
Kochen et al. 2022, Netherlands	To understand the challenges healthcare professionals encounter while providing care for parents during their child’s end-of-life.	Exploratory design	Neonatology and pediatrics in 3 university pediatric hospitals	Thematic analysis	9 (ICU nurse *n* = 1; other *n* = 8)	18/20 (90%), strong
Köktürk Dalcalı, 2022, Turkey	To determine the emotional responses of nurses to work in the neonatal unit and to neonatal deaths.	Descriptive design	NICU of a maternity hospital	Categorical strategy	14	17/20 (85%), strong
Kpassagou 2017, Togo	To explore the emotional strains faced by health practitioners working in a pediatric oncology department.	Qualitative study	Pediatric oncology department of a large teaching hospital	Thematic analysis	16 (pediatric nurse *n* = 9; nurse assistant *n* = 7)	16/20 (80%), good
Laronne et al. 2022, Israel	To explore multi-professional pediatric oncology healthcare communication practices and attitudes to talking to children with advanced disease about their disease, palliative care, and EOL.	Grounded theory	Pediatric cancer centers	Categorical strategy	9	16/20 (80%), good
Laryionava 2018, Germany	To understand oncologists' and oncology nurses’ views on treatment decisions with young adult patients with advanced cancer, (b) to investigate the extent to which young age was a factor in cancer treatment decisions, and (c) to explore possible reasons that may hinder timely decisions and discussions of forgoing treatment in young adult patients.	Grounded theory	Hematology and oncology department of a university hospital	Categorical analysis	7	17/20 (85%), strong
Lewis 2017, USA	To explore the affective, interactional, and meaning-related responses of NICU nurses while caring for dying infants and their families, as well as the coping strategies and changes in practice they experience.	Narrative	NICU	Thematic narrative analysis	36	13/20 (65%), adequate
Lin 2022, Taiwan	To explore the factors leading to and experiences with introducing specialist palliative care to the families of children with life-threatening conditions from the perspectives of pediatric professionals.	Phenomenology	NICU and PICU of a medical center	Secondary, thematic analysis, and theory-driven analysis	7	20/20 (100%), strong
Mitchell 2015, UK	To explore the experiences of senior medical and nursing staff regarding the challenges associated with Advance Care Planning in relation to children and young people with life-limiting illnesses and opportunities for improvement.	Qualitative study	PICU of a tertiary referral center children’s hospital	Thematic content analysis	6	17/20 (85%), strong
Nina 2021, Brazil	To analyze the perception of intensive care doctors and nurses about the death of children.	Qualitative study	PICU, NICU, and CICU of a teaching hospital	Thematic content analysis	7	13/20 (65%), adequate
Özsavran et al. 2024, Turkey	To determine how nurses remembered and made sense of their experiences regarding children’s deaths.	Descriptive and interpretative phenomenological study	PICU of a university hospital	Thematic analysis	13	14/20 (70%), adequate
Shu 2017, Taiwan	To explore the nursing care experience of a preschool-age patient with terminal-stage cancer who faced death, anxiety, and fear.	Case study	Inpatient hospice of a medical center	Narrative analysis	1	13/16 (81%), strong
Stayer and Lockhart 2016, USA	To describe and interpret the essence of the experiences of nurses who provide palliative care to children with life-threatening illnesses and the children’s families.	Hermeneutic phenomenological study	PICU of children’s hospital	Thematic analysis	12	17/20 (85%), strong
Ting 2019, Taiwan	To describe the nursing care experience of an extremely low birth weight premature infant born at 21 weeks and 3 days gestation.	Case study	NICU of a children’s hospital	Narrative analysis	1	13/16 (81%), strong
Ting 2020, Taiwan	To describe the nursing care experience of a pediatrics team working with a sick infant who has the rare genetic disorder trisomy 18, along with the clinical presentations and reflections made during the uncertain process.	Case study	Pediatric team in a teaching hospital	Narrative analysis	1	13/16 (81%), strong
Tsai 2014, Taiwan	To describe the nursing care experience of an 11-year-old child suffering from neuroblastoma using palliative shared care.	Case study	Pediatric care team of a medical center	Narrative analysis	1	13/16 (81%), strong
Tseng 2019, Taiwan	To describe the nursing care experience of a teenager with Rhabdomyosarcoma.	Case study	Pediatric oncology ward of a medical center	Narrative analysis	1	13/16 (81%), strong
Willis 2019, USA	To explore experience of perinatal loss from the perspective of the nurse.	Qualitative descriptive design	2 hospitals	Content analysis	9	12/20 (60%), adequate
Yu 2022, Taiwan	To describe the nursing care experience of a pre-teen patient who suffer from malignant sarcoma and lung metastasis at his end-of-life stage.	Case study	Pediatric oncology ward of a medical center	Narrative analysis	1	14/16 (88%), strong
Zhang et al. 2022, China	To explore nurses’ experiences coping with patient death.	Qualitative study	Emergency ward, oncology department, and ICU of 2 tertiary public hospitals	Inductive thematic analysis	15 (emergency ward nurse *n* = 5; oncology nurse *n* = 6; ICU nurse *n* = 4)	18/20 (90%), strong

NICU = neonatal intensive care unit; PICU = pediatric intensive care unit.

a Information on the relevant data source and sample included in the review.

### Theme 1: Never seem to feel fully prepared

A child’s complex needs stress both families and nurses. Nurses frequently mentioned frustration about their lack of experience in caring for cases of life-limiting or life-threatening conditions due to the rarity of care guidance and resources for families (Gilleland et al. 2014; Donohue et al. 2018), as well as the unpredictability of a patient’s condition (Chew et al. 2021). There was vagueness in pediatric palliative care “to define what the exact needs are” (Burgers et al. 2022), or “don’t have enough staff to safely deliver the care” (Constantinou et al. 2024) may further cause “hesitancy and uncertainty” around how to help patients and families (Avery et al. 2020). Additionally, some nurses find that palliative care's emphasis on private rooms for families conflicts with the traditional nursing culture of having a “backup” system, where “nurses were able to see and hear all of the patients, monitor multiple patients at the same time,” and that “there was always someone who could help you with something” (Coats et al. 2018). Some nurses also felt the pressure of being constantly observed by families at bedside (Coats et al. 2018).

Some nurses had experience that families’ behavior often changes to the “fighting” stance when families experience resistance in accessing what they need, or even used the phrase “shout the loudest” to describe their behavior to obtain what they need for their children, especially for those children who had been cared for by the families over a long time (Constantinou et al. 2024). As a result, these conditions bring nurses a sense of failure and helplessness, especially when the patient experiences anxiety, fear, and pain (Hsu and Liu, 2014), or when the patient is “critically stress” and the family members “are physically and verbally threatening” (Coats et al. 2018; Laronne et al. 2022).

The father came with a camera and shoved it up everybody’s nose and took photos of everybody. They were both screaming “murderers, murderers” at everybody, photographing the equipment, photographing the monitors, none of the nursing staff wanted to work there, the level of stress was enormous…… [The father] physically pushed the nurses. (PICU consultant; Forbat et al. 2015)

Nurses have a unique role in assisting family members in carrying out caregiving activities by providing the necessary skills and knowledge. When a child is dying, nurses are expected to be well-prepared by knowing “what to do and what to say to the parents and family” (Almeida et al. 2016; Chew et al. 2021), cognitively to be aware of the child’s deteriorating condition (Bassola et al. 2023), and to develop a perception that “there were sacrifices…(for) whatever life expectancy you’re losing” (De Abreu Haickel Nina et al. 2021; Deschenes et al. 2024).

However, nurses themselves also expressed their struggles in understanding the situation, feeling disoriented, nervous, scared, anxious, panicked, confused, and blank, “don’t have a guideline,” especially when a child passed away (Pearson 2013; Willis 2019; Chew et al. 2021; Bassola et al. 2023). Some nurses mentioned that they “don’t know how to start”, “how to do”, and “never seem to feel fully prepared” in the situation of supporting the family or alleviating the child’s symptoms (Pearson 2013; Hsu and Liu, 2014; Almeida et al., 2016).

I think the struggle comes when you want to make [the family] feel better, yet at the same time, you… you know, there is a balance between making them feel balanced and not knowing how to make them feel better… (New nurse in a women’s and children’s hospital)

Moreover, when it comes to end-of-life decision making, a lack of advanced care planning with families can also cause nurses to be insecure in their doing and acting (Lotz et al. 2015). When nurses have difficulties in making sense of their role in a dying child, they would often feel stressed when they were committed to be “there to whatever they might need” (Almeida et al. 2016; Stayer and Lockhart 2016), “try as much as possible to comfort the loss” of the families (Medeiros et al. 2022), or to “offer the help” even knowing that their “best effort is not enough” (Burgers et al. 2022; Özsavran et al. 2024). Emphasizing the feeling of discouragement and professional failure that follows poor treatment and palliative care outcomes, nurses often feel that they were “working for nothing” (Kpassagou and Soedje 2017). This led them to question the care they provided and feel like they failed their task as a nurse (Al Mutair et al. 2019).

### Theme 2: Sense of unfairness in conflicts of values

Feelings of “unfairness” appear from multiple causes when nurses’ values are at odds with those of patients, families, and other healthcare professionals. Most often, these feelings were related to the delivery of futile care or the inability to help a patient survive (Lewis 2017; Laryionava et al. 2018). Nurses articulated their concerns when they perceived that seriously ill neonates would have “absolutely no good quality of life” (Bassola et al. 2023) or when caring for chronic patients in the intensive care unit (ICU) who had “no planned or foreseeable exit route” (Geoghegan et al. 2016). However, nurses sometimes struggle with overvaluing physicians’ opinions and “undervaluing trusting or expressing their own instincts” (Banazadeh et al. 2021; Medeiros et al. 2022). They often experience mental distress when they are unsatisfied with physicians’ therapeutic plans or parents’ decisions, yet are unable to take effective actions (Pearson 2013; Lewis 2017). For example, nurses frequently consider that the treatment should be withdrawn to minimize distress to the child, yet the physician or families did not understand, accept, and participate in the palliative care program planned, and are continuing to “want all interventions possible” (Pearson 2013).

These conflicts are especially difficult when the patient is experiencing suffering, “who depends on us [nurses] and the machines for everything, who does not react, has no contact with the outside world” (Bassola et al. 2023). Many nurses grappled with feelings of guilt, expressing sentiments such as “we feel like we’re torturing him [the patient]” (Deschenes et al. 2024). This generated a challenging environment “where no one feels that they’ve contributed to anybody’s wellbeing” (Geoghegan et al. 2016). Ultimately, this dialogue encourages nurses to reflect on the profound impact of their dedication and the critical question of how deeply they should invest in sustaining the patients’ lives.

Nurses described expecting that they could be truthful to family members and “not to generate an expectation that the staff know will not exist” (Medeiros et al. 2022). However, the phenomenon of “physicians’ loyalty” on the part of families could sometimes make nurses feel ethical conflict and unfairness because it “prevents physicians from telling the truth” to family members (Banazadeh et al. 2021; Medeiros et al. 2022). Additionally, nurses who acted independently of other team members often experienced a feeling of “isolation” and caused emotional discomfort in the rest of the team members (Fernández-Alcántara et al. 2020). If the nurses actively provided information for the families that contradicted the physician, this would make families “furious” (Banazadeh et al. 2021).

The SiPAP [Synchronous Inspiratory Positive Airway Pressure] and IV fluids were stopped [as methods of discontinuing life support] … I did not agree that the family had the right to stop the IV fluids…. This baby essentially died of dehydration, and we, as hospital staff, assisted in killing her…. (NICU nurse, Lewis 2017)

Nurses also reported having painful feelings when they are reluctant or don’t feel comfortable when the patient transitions into palliative care, and that they have to discontinue therapeutic intervention (Medeiros et al. 2022). When nurses’ practices are not based on their ethical norms, they may have moral distress, particularly as their feelings of advocacy are strong but fail to speak for the patients and families (Lewis 2017), or when they have difficulty in taking an active role to change conditions that they perceive as unethical.

### Theme 3: Sheathing against traumatic bond attachment

Some nurses described that they end up getting involved, creating a very great bond: family, patient, and staff, until the child died (Almeida et al. 2016). However, in many cases, nurses establish “emotional boundaries,” “creating defense mechanisms” and “become more selfish” in advance for “keeping the gap” to “protect” themselves, and to “cope,” keeping themselves from building close relationships with patients and family to protect themselves from suffering (Pearson 2013; Almeida et al. 2016; Atout et al. 2019; Banazadeh et al. 2021; Duarte et al. 2021; Bassola et al. 2023; Özsavran et al. 2024).

In some cases, nurses experienced negative feelings when they compared their own lives with those of the patient and families, and found that their life is “good” (Lewis 2017). There “was a sense of relatedness” that occurs when nurses were in or around the young adults’ age, attributed this to the fact that they are in the same life phase and sometimes even can identify with the young adults and their family, “a connection” that increased the burden of care (Kpassagou and Soedje 2017; Avery et al. 2020; Burgers et al. 2022). Some nurses mentioned feelings of grief were associated with having healthy children, less tragedy in their own lives, especially in pregnant NICU nurses. The traumatic grief and loss following such relatedness may be aware or unaware. Some nurses remain unaware of the extent of their efforts while providing care to dying children. After losing these children, they would feel physically “exhausted,” including “stress,” “fatigue,” “headache,” and “not rested” (Willis 2019; Özsavran et al. 2024). While others who are fully aware of the traumatic experiences stated that these “will never leave” (Lewis 2017).

“… the memory of doing chest compressions on this big baby while carrying my own baby will never leave me ….” (NICU nurse; Lewis 2017)

Nurses’ manner of portraying the emotion included such descriptors as “overwhelming,” “horrendous,” “burnout,” “dropped in to someone else’s nightmare” to “suffer together with the family” in this “shared grief” process (Mitchell and Dale 2015; Lewis 2017; Donohue et al. 2018; Al Mutair et al. 2019; Chew et al. 2021; Devitt and Hara 2021; Duarte et al. 2021; Kochen et al. 2022; Köktürk Dalcali et al. 2022; Özsavran et al. 2024). They even did “not want to memorize” previous experiences or “to think about the story of these children” for the sake of creating a detachment (Willis 2019; Bassola et al. 2023). One of the reasons described was that nurses felt like “regardless of how hard you try, there’s no way that you can meet the needs of that family” (Chen et al. 2015; Donohue et al. 2018). These emotional burdens would negatively affect nurses’ work functioning; therefore, instead of getting deeper into a multidisciplinary need, nurses were convinced to focus only on the “needs in the present,” to become “task focused” (Willis 2019; Bassola et al. 2023), and to “push yourself to be strong” (Al Mutair et al. 2019; Duarte et al. 2021).

It’s not real life what’s going on in there, it’s just so horrendous what is happening every single day … so ‘No, you’re not doing it today. You’ve done it a couple of times recently, and that is enough.’ Because if you do it too often, you have to leave. You have to protect yourself. (PICU nurse; Mitchell and Dale 2015)

In some cases, nurses passively described themselves as “I did what I could, I couldn’t do more,” “we can’t do anything” (Banazadeh et al. 2021; Bassola et al. 2023). Others stated that this was due to “the lack of medical supplies and equipment to provide quality care” (Kpassagou and Soedje 2017). Avoiding a dying patient, at the same time, “normalizing and accepting death as part of life” appears to be a common coping strategy for nurses. ICU nurse stated that it was quite “protective” of staff of the short-term nature of ICU “because then they don’t often get to know children and families very well” (Kpassagou and Soedje 2017). Some nurses adjust their perceived obligation and appraise the quality outcome of their care based on a lower standard. For example, nurses often preferred to believe that these children did not feel pain, at least when they were dying or after their death, or that they did not hurt the patients; otherwise, it would make the care process more difficult (Medeiros et al. 2022; Özsavran et al. 2024).

In other cases, families refuse the interference of nurses providing palliative care, described as not wanting them to “interfere with anything” or not wanting them “to get in the middle of their world.” This rejection not only makes it difficult for nurses to be “sympathetic” to the families but also causes more avoidance of nurses to build bonds with them (Almeida et al. 2016; Bassola et al. 2023).

When I see that a child has cancer in an advanced stage… when I expect that the child will die, my relationship with him or her is less close. (Nurse in the pediatric oncology department of a hospital; Kpassagou and Soedje 2017)

I was grieving along with the parents and came in often, even when not on my shift, to be with them… I had crossed some professional boundaries, but at the time felt compelled to be there for the parents and myself. (NICU nurse; Lewis 2017)

## Discussion

This study identified 3 themes that illuminate the emotional stress nurses experience when addressing the needs of families: never seem to feel fully prepared, sense of unfairness in conflicts of values, and sheathing against traumatic bond attachment. Together, these themes reflect the moral, emotional, and relational tensions embedded in pediatric palliative care. As Burkhardt and Nathaniel (2020) contend, nursing is fundamentally oriented toward fulfilling health-related needs, thereby creating a professional responsibility to enhance pediatric patients’ quality of life while supporting families as they adapt to the profound grief associated with the loss of a child. These societal and professional expectations generate a strong moral imperative for nurses to respond simultaneously to the needs of patients and families, often under conditions of uncertainty and emotional intensity.

Despite this responsibility, ambiguity in the definition and implementation of pediatric palliative care continues to blur professional boundaries within nursing practice. This lack of role clarity may contribute to feelings of being overwhelmed, a diminished sense of control, and ultimately a perception of being unsafe in the workplace (Bamforth et al. 2023). When expectations exceed clearly articulated roles and available support structures, nurses are left to navigate ethically complex situations with insufficient guidance, thereby increasing their vulnerability to moral distress.

Nurses’ experiences of providing potentially futile care in palliative and end-of-life contexts are further shaped by the interaction of culturally mediated meanings and organizational power structures (Choi et al. 2024; Shhadat et al. 2025). For nurses from certain minority or religious communities, continuous support may be understood as an expression of duty, hope, the sanctity of life, or respect for family and community expectations, rather than simply as “non-beneficial care.” Yet not all spiritual traditions endorse unrestricted intervention; recent research on Islamic end-of-life care, for instance, highlights that comfort, dignity, and cessation of futile treatment can be morally acceptable when decisions are made through family-centered and faith-informed approaches (Chamsi-Pasha and Albar 2017). Concepts such as hope, suffering, parental obligation, and the meaning of a “good death” are interpreted differently by families and physicians, shaping whether continued life-sustaining treatment is viewed as burdensome overtreatment or as morally meaningful preservation of life (Choi et al. 2024). These tensions are particularly pronounced in pediatric settings, where prognostic uncertainty and intense parental grief complicate goals-of-care discussions and heighten emotional and ethical stakes (Begjani et al. 2022).

However, nurses’ moral distress should not be attributed solely to cultural differences or framed as a “cultural issue” (Chamsi-Pasha and Albar 2017). Growing evidence indicates that moral distress is more strongly associated with structural and relational constraints, including poor nurse-physician collaboration, weak nurse autonomy, hierarchical decision-making, and inadequate interprofessional communication (Begjani et al. 2022; Rezaei et al. 2023). In hierarchical healthcare cultures, doctors may retain the final authority while nurses remain ethically accountable for patient suffering at the bedside but are structurally powerless to influence treatment objectives. These systemic conditions compel nurses to continue interventions they personally judge to be non-beneficial, contributing to cumulative moral distress and reinforcing feelings of helplessness and moral residue over time.

In addition to organizational and ethical challenges, nurses face significant personal stressors related to the intersection of professional and familial roles. Consistent with previous studies, the simultaneous responsibility of caring for pediatric patients with life-limiting conditions while managing one’s own parental duties constitutes a substantial emotional burden. As Bamforth et al. (2023) observe, nurses often prioritize patient care over their own needs, resulting in the suppression of their psychological well-being. Over time, this pattern may perpetuate emotional exhaustion, particularly in high-intensity environments characterized by sustained emotional labor. More research is needed to explore how caring for terminally ill pediatric patients influences nurses’ own parenting experiences and decisions related to motherhood.

The emotional attachments nurses form with patients and families represent another significant source of stress. Betan et al. (2005) noted that negative countertransference reactions, such as feelings of being overwhelmed, disorganized, helpless, or inadequate, often reflect a combination of the nurse’s personal dynamics, responses triggered by patients and their families, and the overall interaction between the patients, families, and the nurse. Prolonged engagement in emotionally intense relationships can lead to cumulative grief and distress, supporting evidence that excessive emotional investment contributes to burnout (Xie et al. 2021). When nurses feel emotionally overwhelmed and unable to provide the level of care they strive for, they may resort to emotional detachment as a coping mechanism (Bakker and Demerouti 2017; Atout et al. 2019; Bamforth et al. 2023). Although detachment may provide temporary relief, it can compromise care quality and negatively affect professional functioning, underscoring the importance of institutional mechanisms that support emotional processing and resilience.

To address these challenges more coherently, the cultural dimension of pediatric palliative care is more effectively conceptualized through a shift from cultural competence to cultural humility (Singh et al. 2023). Unlike cultural competence, which emphasizes the acquisition of discrete knowledge about cultural groups, cultural humility is an ongoing practice grounded in reflexivity, openness, and deliberate efforts to address power imbalances within clinical encounters and healthcare institutions (Foronda et al. 2016). This approach is particularly relevant in ethically complex palliative and end-of-life decisions, where deeply held family values must be engaged without stereotyping and where clinical authority may inadvertently marginalize nursing perspectives (Salahshurian and Moore 2024).

Furthermore, nurses must cultivate strong collaborative capacities within multidisciplinary teams. Such collaboration begins with an awareness of one’s own values and emotional responses to ethically challenging situations. Recognizing personal values alongside those of colleagues, patients, and families enables nurses to engage in reflective, value-informed decision-making rather than relying on instinctive responses shaped by prior experiences. Bamforth et al. (2023) further emphasize that nurses often require explicit organizational and cultural permission to prioritize self-care, making the identification and implementation of individualized stress management strategies essential. Meaningful relationships with families, particularly when appreciation and positive feedback are expressed, may further enhance nurses’ sense of professional fulfillment (Almeida et al. 2016). Building on this foundation, it is important to normalize awareness of countertransference, strengthen access to structured debriefing and reflective supervision, and explicitly provide nurses with protected opportunities for self-care and recovery. Such opportunities may include peer support, mindfulness practices, counseling access, and rotation away from high-intensity cases when feasible. Ultimately, fostering emotional resilience, supportive relational networks, and a culture that values self-care is critical for sustaining nurses’ well-being amid the ethical and emotional demands inherent in pediatric palliative care.

## Strengths and limitations

This study broadly included case studies of Asian nurses who had fewer opportunities to voice their experiences in this dominant Western worldview. It would be worthwhile to expand future research to explore nurses’ stress in various settings, socioeconomic status, age, size of their network, and so on. By addressing both families’ needs and nurses’ well-being simultaneously, we can create more sustainable and compassionate pediatric palliative care systems that benefit all stakeholders involved in this challenging but vital area of healthcare.

## Conclusions

This meta-synthesis provides a deeper and more integrated understanding of nurses’ experiences in meeting the complex needs of families within pediatric palliative care. By synthesizing nurses’ accounts across studies, this research highlights how emotional strain, coping challenges, and moral tensions are embedded within nurses’ efforts to fulfill professional responsibilities amid profound family suffering. The findings reveal not only the intensity of psychological burden nurses carry, but also the conditions under which coping may fail, contributing to emotional withdrawal and, for some, disengagement from palliative and end-of-life care practice. These experiences underscore how role ambiguity, interpersonal conflicts, and sustained emotional labor intersect to shape nurses’ capacity to remain engaged in this field. Gaining insight into these stressors advances understanding of the relational and ethical complexities inherent in pediatric palliative care and draws attention to the need for greater recognition of nurses’ emotional labor. A clearer appreciation of these dynamics is essential for developing supportive structures that address psychological well-being, sustain professional engagement, and ultimately enhance the quality of care provided to children and their families.

## Supporting information

10.1017/S1478951526102880.sm001 Priyanti et al. supplementary material Priyanti et al. supplementary material

## Data Availability

Data will be made available on request.

## Supplementary material

The supplementary material for this article can be found at https://doi.org/10.1017/S1478951526102880.

## Figure 1 Long description

The flowchart illustrates the PRISMA process for selecting studies. It begins with 'Identification' where records are identified from databases totaling 1,841, including Airiti Library (178), CINAHL (175), Cochrane Library (5), Embase (432), Ovid Medline (946) and PubMed (105). Duplicates removed before screening are 108. Additionally, 1 record is identified from citation searching. In 'Screening', 1,733 records are screened, with 1,586 excluded. Reports sought for retrieval are 147, with none not retrieved. Reports assessed for eligibility are 147, with exclusions due to incorrect population (30), incorrect intervention (24), incorrect context (18), review article (8), only abstract (20) and not clearly labeled as a nurse (2). Finally, 46 studies are included in the review.

Navigate back to Figure 1.

## Table 1 Long description

The table summarizes 40 included studies by author, year, country, aim, study design, clinical setting, analysis method, nurse sample size, and quality appraisal score. Most studies are qualitative (descriptive, phenomenology, grounded theory, or exploratory) using thematic or content-based analyses; a smaller set are case studies using narrative analysis. Settings cluster in neonatal and pediatric intensive care, pediatric oncology, and pediatric wards, with some multi-hospital or community-linked services. Nurse participant samples range from single-nurse case reports to 36 nurses, with many studies in the single digits to teens and several around 10 to 20 participants. Quality ratings are predominantly good or strong, with several perfect scores reported and a smaller number rated adequate. Countries represented are diverse, with multiple studies from Taiwan and additional studies from Brazil, the United States, the United Kingdom, and other regions. Because the table compiles heterogeneous aims and methods, quality scores and sample sizes should be compared cautiously across different study designs and appraisal scales.

Navigate back to Table 1.
