# Supplementary material for: Nurses’ stressful experiences in providing palliative and end-of-life care for children: A meta-synthesis
Source: Palliat Support Care. 2026 Jul 20;24:e191. doi: 10.1017/S1478951526102880 (PMC13430483; doi:10.1017/S1478951526102880)
Supplement: Priyanti et al. supplementary material [file S1478951526102880sup001.docx]

Appendix-1

Medline Search Strategy

| Number | Searches | Results |
| --- | --- | --- |
| S1 | (Adolescen* or Bab* or Child or Infant or Juvenile or Minor* or P*ediatric or Preschool or "Prime adult*" or Schoolchild or Teen* or (Young adj (adult* or people or person*)) or Younger or Youth or Perinatal or Neonatal).mp | 266,597 |
| S2 | (exp Adolescent/ or exp Adult children/ or exp Child/ or exp Infant/ or exp Minors/ or exp Paediatrics/ or exp Young adult/ or exp Perinatal death/) | 71,322 |
| S3 | ((Advanced adj (cancer* or illness*)) or "Catastrophic ill*" or "Critical* ill*" or Deteriorat* or Dying or "End of life" or End-of-life or (Life adj (limit* or threaten*)) or Life-limit* or Life-threaten* or "Rare disease*" or Terminal* or "end stage").mp | 10,528 |
| S4 | exp Catastrophic illness/ or exp Chronic disease/ or exp Critical illness/ | 19,916 |
| S5 | (Palliative* or Hospice* or "Supportive care").mp | 2,102 |
| S6 | exp Hospice and palliative care nursing/ or exp Hospices/ or exp Palliative care/ | 474 |
| S7 | (nurse or nurses or nursing or staff).mp | 7,836 |
| S8 | exp nurses/ or exp personnel, hospital or exp health educators or exp nursing staff/ or exp faculty, nursing/ or exp case managers/ or exp nursing assistants/ | 1,875 |
| S9 | depress* or "mental health" or anxiety* or anxious* or burnout | 1,325,417 |
| S10 | exp anxiety disorders/ or exp depression/ or exp depressive disorder/ exp burnout, professional/ | 9,568 |
| S11 | S1 or S2 | 337,396 |
| S12 | S3 or S4 or S5 or S6 | 32,547 |
| S13 | S7 or S8 | 9,693 |
| S14 | S9 or S10 | 1,325,417 |
| S15 | S11 and S12 and S13 and S14 | 1,384 |
| S16 | limit S15 to year="2014-2025" | 1,011 |
| S17 | limit S16 to "human" | 948 |
| S18 | limit S17 to "English" | 946 |

Embase Search Strategy

| Number | Searches | Results |
| --- | --- | --- |
| 1 | (Adolescen* or Bab* or Child or Infant or Juvenile or Minor? or P?ediatric or Preschool or "Prime adult?" or Schoolchild or Teen* or (Young adj (adult? or people or person?)) or Younger or Youth or Perinatal or Neonatal):ti,ab,kw,de | 5,653,613 |
| 2 | Child/exp or Adolescent/exp or "Young adult"/exp or "Minor (person)"/exp or Paediatrics/exp or Preschool/exp | 5,818,151 |
| 3 | ((Advanced adj (cancer* or illness*)) or "Catastrophic ill*" or "Critical* ill*" or Deteriorat* or Dying or "End of life" or End-of-life or (Life adj (limit* or threaten*)) or Life-limit* or Life-threaten* or "Rare disease*" or Terminal*):ti,ab,kw,de | 1,566,653 |
| 4 | "Advanced cancer"/exp or "Chronic disease"/exp or "Critical illness"/exp or Deterioration/exp or Dying/exp or "End of life"/exp or "Rare disease"/exp or "Terminal disease"/exp or "perinatal death"/exp or "child death"/exp | 1,047,563 |
| 5 | (Palliative* or Hospice* or "Supportive care"):ti,ab,kw,de | 283,058 |
| 6 | Hospice/exp or "Palliative therapy"/exp or "Palliative nursing"/exp or "Supportive care"/exp | 260,326 |
| 7 | (nurse or nurses or nursing):ti,ab,kw,de | 1,016,753 |
| 8 | nurse/exp | 486,492 |
| 9 | (depress* or "mental health" or anxiety* or anxious* or burnout):ti,ab,kw,de | 1,804,721 |
| 10 | depression/exp or emotion/exp or "mental stress"/exp | 1,923,388 |
| 11 | 1 or 2 | 6,567,768 |
| 12 | 3 or 4 or 5 or 6 | 2,361,203 |
| 13 | 7 or 8 | 1,048,638 |
| 14 | 9 or 10 | 2,413,497 |
| 15 | 11 and 12 and 13 and 14 | 3,723 |
| 16 | 15 limit to “human” | 3,602 |
| 17 | 16 and ('grounded theory'/de OR 'interview'/de OR 'qualitative research'/de OR 'semi structured interview'/de OR 'telephone interview'/de) | 494 |
| 18 | 17 and year 2014-2025 | 437 |
| 19 | 18 and ([chinese]/lim or [english]/lim) | 432 |

Pubmed Search Strategy

| Number | Searches | Results |
| --- | --- | --- |
| 1 | (Adolescen*[tw] or Bab*[tw] or Child[tw] or Infant[tw] or Juvenile[tw] or Minor*[tw] or P*ediatric[tw] or Preschool[tw] or "Prime adult*"[tw] or Schoolchild[tw] or Teen*[tw] or (Young[tw] and (adult*[tw] or people[tw] or person*[tw])) or Younger[tw] or Youth[tw] or Perinatal[tw] or Neonatal[tw]) | 5,872,075 |
| 2 | Adolescent[mh] or Adult children[mh] or Child[mh] or Infant[mh] or Minors[mh] or Paediatrics[mh] or Young adult[mh] or Perinatal death[mh] | 4,743,144 |
| 3 | (Advanced[tw] and (cancer*[tw] or illness*[tw])) or "Catastrophic ill*"[tw] or "Critical* ill*"[tw] or Deteriorat*[tw] or Dying[tw] or "End of life"[tw] or End-of-life[tw] or (Life[tw] and (limit*[tw] or threaten*[tw])) or Life-limit*[tw] or Life-threaten*[tw] or "Rare disease*"[tw] or Terminal*[tw] or end stage[tw] | 1,566,262 |
| 4 | Catastrophic illness[mh] or Chronic disease[mh] or Critical illness[mh] | 712,616 |
| 5 | Palliative*[tw] or Hospice*[tw] or "Supportive care"[tw] | 152,054 |
| 6 | Hospice and palliative care nursing[mh] or Hospices[mh] or Palliative care[mh] | 73,100 |
| 7 | nurse[tw] or nurses[tw] or nursing[tw] or staff[tw] | 1,036,058 |
| 8 | (nurses[mh] or personnel, hospital[mh] or health educators[mh]) or nursing staff[mh] or faculty, nursing[mh] or case managers[mh] or nursing assistants[mh] | 230,105 |
| 9 | depress*[tw] or "mental health"[tw] or anxiety*[tw] or anxious*[tw] or burnout[tw] | 1,176,670 |
| 10 | anxiety disorders[mh] or depression[mh] or depressive disorder[mh] or burnout, professional[mh] | 376,894 |
| 11 | 1 or 2 | 5,890,047 |
| 12 | 3 or 4 or 5 or 6 | 2,265,601 |
| 13 | 7 or 8 | 1,056,968 |
| 14 | 9 or 10 | 1,210,022 |
| 15 | 11 and 12 and 13 and 14 | 1,641 |
| 40 | limit 39 to humans | 1,485 |
| 41 | limit 40 to (chinese or english) | 1,409 |
| 42 | limit 41 to yr="2014 - 2025" | 839 |
| 44 | limit 43 to Interview or Review or Systematic review | 105 |

CINAHL Search Strategy

| Number | Searches | Results |
| --- | --- | --- |
| 1 | (Adolescen* or Bab* or Child or Infant or Juvenile or Minor* or P*ediatric or Preschool or "Prime adult*" or Schoolchild or Teen* or (Young and (adult* or people or person*)) or Younger or Youth or Perinatal or Neonatal) | 8,317,740 |
| 2 | (Advanced and (cancer* or illness*)) or "Catastrophic ill*" or "Critical* ill*" or Deteriorat* or Dying or "End of life" or End-of-life or (Life and (limit* or threaten*)) or Life-limit* or Life-threaten* or "Rare disease*" or Terminal* or end stage | 340,694 |
| 3 | Palliative* or Hospice* or "Supportive care" | 93,475 |
| 4 | nurse or nurses or nursing or staff | 1,163,415 |
| 5 | depress* or "mental health" or anxiety* or anxious* or burnout | 510,060 |
| 6 | 2 or 3 | 390,053 |
| 7 | 1 and 4 and 5 and 6 | 6,099 |
| 8 | limit 7 to humans | 4,100 |
| 9 | limit 8 to (chinese or english) | 3,808 |
| 10 | limit 9 to yr="2014-2025" | 2,652 |
| 11 | limit 10 to qualitative studies | 1,922 |
| 12 | limit 11 to Infant, Newborn: birth-1 month, Infant: 1-23 months, Child, Preschool: 2-5 years, Child: 6-12 years, Adolescent: 13-18 years | 204 |
| 13 | exclude Medline records | 175 |

The Cochrane Library Search Strategy

| Number | Searches | Results |
| --- | --- | --- |
| 1 | (Adolescent or baby or child or infant or juvenile or minor or pediatric or preschool or "prime adult" or schoolchild or teen or "young adult" or "young people" or "young person" or younger or youth or perinatal or neonatal):ti,ab,kw | 417,714 |
| 2 | MeSH descriptor: [Adolescent] explode all trees | 138,148 |
| 3 | MeSH descriptor: [Child] explode all trees | 83,147 |
| 4 | MeSH descriptor: [Infant] explode all trees | 46,120 |
| 5 | MeSH descriptor: [Pediatrics] explode all trees | 1,067 |
| 6 | MeSH descriptor: [Perinatal Death] explode all trees | 164 |
| 7 | ("Advanced cancer" or "advanced illness" or "catastrophic ill" or "critical ill" or deteriorate or dying or "end of life" or end-of-life or "life limited" or "life threatened" or "life-limiting" or "life-threatened" or "rare disease" or terminal):ti,ab,kw | 32,236 |
| 8 | MeSH descriptor: [Terminally Ill] explode all trees | 122 |
| 9 | (Palliative or Hospice or "supportive care"):ti,ab,kw | 19,425 |
| 10 | MeSH descriptor: [Palliative Care] explode all trees | 2,655 |
| 11 | MeSH descriptor: [Hospice Care] explode all trees | 181 |
| 12 | MeSH descriptor: [Bereavement] explode all trees | 377 |
| 13 | MeSH descriptor: [Terminal Care] explode all trees | 774 |
| 14 | (nurse or nurses or nursing):ti,ab,kw | 84,550 |
| 15 | MeSH descriptor: [Nurses] explode all trees | 4,417 |
| 16 | MeSH descriptor: [Nursing Staff] explode all trees | 907 |
| 17 | MeSH descriptor: [Case Managers] explode all trees | 937 |
| 18 | (depression or "mental health" or anxiety or anxious or burnout):ti,ab,kw | 186,156 |
| 19 | MeSH descriptor: [Depression] explode all trees | 19,523 |
| 20 | MeSH descriptor: [Anxiety] explode all trees | 13,616 |
| 21 | MeSH descriptor: [Burnout, Psychological] explode all trees | 735 |
| 22 | MeSH descriptor: [Stress, Psychological] explode all trees | 9,655 |
| 23 | 1 or 2 or 3 or 4 or 5 or 6 | 417,741 |
| 24 | 7 or 8 or 9 or 10 or 11 or 12 or 13 | 48,155 |
| 25 | 14 or 15 or 16 or 17 | 85,554 |
| 26 | 18 or 19 or 20 or 21 or 22 | 191,115 |
| 27 | 23 and 24 and 25 and 26 | 494 |
| 28 | limit 27 to date from Jan 2024 to Sep 2025 | 65 |
| 29 | limit 28 to Qualitative studies | 5 |
